# Effect of ALA on preventing diabetic peripheral neuropathy in rats through mitochondrial axonal transport

**DOI:** 10.1371/journal.pone.0346297

**Published:** 2026-04-01

**Authors:** Jiaxin Tian, Jingwen An, Linchun Song, Wang Zhang, Die Chen, Tianya Zhang, Ying Ben

**Affiliations:** 1 Hebei University of Chinese Medicine, Shijiazhuang, China; 2 Hebei Key Laboratory of Integrative Medicine on Liver–Kidney Patterns, Shijiazhuang, China; Advanced Materials Technology Research Institute, National Research Centre, EGYPT

## Abstract

**Purpose:**

To explore the mechanism by which alpha-lipoic acid (ALA) regulates mitochondrial axonal transport to protect the sciatic nerve in diabetic rats.

**Methods:**

Among 55 healthy male Sprague‒Dawley rats, 40 were randomly selected, fed a high-carbohydrate/high-fat diet and intraperitoneally injected with streptozotocin (STZ) to induce diabetes. Diabetic rats were randomly divided into diabetic peripheral neuropathy (DPN) and alpha lipoic acid (ALA) groups, with 15 rats in each group, excluding the rats in which diabetes failed to be induced and the dead rats. The other 15 rats were used as the control group. The rats in the ALA group were administered an ALA suspension (60 mg/kg/day) by gavage for 12 weeks. The rats in the control group and DPN group were gavaged with an equal volume of distilled water every day for 12 weeks. After the intervention was complete, the motor nerve conduction velocity (MNCV) and paw withdrawal threshold (PWT) were measured. Morphological changes in the sciatic nerve were observed by HE staining. Immunofluorescence staining and Western blotting were performed to measure protein expression levels. In vitro, a model of NSC34 cell injury was established by treating cells with high concentrations of glucose and palmitic acid sodium. NSC34 cells were randomly divided into the control group, DPN group and ALA group. The ALA group was treated with ALA for 24 hours. Changes in the axons of NSC34 cells were assessed by measuring the length of the axons. Immunofluorescence staining was performed to determine the fluorescence intensity of the cells, and Western blotting was performed to determine the grayscale value of each band.

**Results:**

ALA increased the MNCV and PWT in DPN rats and increased the levels of phosphorylated AMP-activated protein kinase (p-AMPK) and phosphorylated cAMP-response element-binding protein (p-CREB). The expression of a motor protein involved in anterograde axonal mitochondrial transport, kinesin family member 5A (KIF5A), was upregulated, whereas the expression of a dynein protein involved in mitochondrial retrograde transport, Dynein cytoplasmic 1 intermediate chain 2 (DYNC1I2), was downregulated by ALA.

**Conclusion:**

The results of this study suggest that ALA alleviates peripheral nerve injury in diabetic rats by promoting the anterograde axonal transport of mitochondria, which may be related to AMPK/CREB signaling.

## 1. Introduction

Diabetic peripheral neuropathy (DPN) is the most common complication of diabetes mellitus (DM) and is a leading cause of disability. The most common form of DPN is distal symmetrical multiple peripheral neuropathy, which primarily affects sensory, motor, and autonomic nerves [1]. In severe cases, DPN can result in intense pain and even the need for amputation, significantly impacting the quality of life of patients [2].

Peripheral nerve demyelination and axonal degeneration are the main pathological characteristics of DPN [3]. The survival of peripheral nerve axon ends relies heavily on energy support. Because neurons have a high energy demand and unique polarized structure, mitochondrial axonal transport is required to maintain energy homeostasis throughout these cells, especially in distal axons [4]. Neurons utilize complex transport mechanisms to transport healthy mitochondria, which act as local energy sources, to axon terminals and remove damaged mitochondria from the distal region to maintain axonal bioenergetics [5]. Neurons maintain energy homeostasis in axons through mitochondrial axonal transport. One of the motor proteins involved in anterograde axonal mitochondrial transport is kinesin family member 5A (KIF5A), which is crucial for maintaining the survival and function of neurons [6]. Dynein is crucial for the retrograde transport of mitochondria in the axons of neurons. Its main function is to transport aging or damaged mitochondria from the distal end of axons to the neuron cell body in a timely manner [7]. Dynein cytoplasmic 1 intermediate chain 2 (DYNC1I2) is the intermediate chain of dynein.

The axonal transport of mitochondria responds to changes in biological energy status as neurons grow and become damaged, ensuring an adequate supply of ATP at these metabolically active locations. AMP-activated protein kinase (AMPK) plays a crucial role in maintaining cellular energy balance and is involved in various physiological activities [8]. Activation of AMPK can lead to the activation of cAMP-response element-binding protein (CREB) and increase its transcription. CREB is phosphorylated by AMPK. Studies have shown that AMPK activation leads to CREB phosphorylation, and when AMPK is inhibited, CREB expression also decreases [9,10]. CREB, an important nuclear transcription factor, can upregulate KIF5A, thereby promoting anterograde mitochondrial transport [11]. Dysregulation of AMPK signaling and impaired axonal energy metabolism occur in certain neurodegenerative diseases [12]. However, studies investigating the effect of AMPK on the sciatic nerve during the development of DPN are limited.

DPN affects nerves in a length-dependent manner. For example, fatty acids have important effects on mitochondrial axonal transport [13]. Early neuronal dysfunction in DPN often manifests distal to the axon. This process is closely related to the importance of mitochondrial axonal transport in ensuring the proper delivery of mitochondria from the cell body to axons and synapses, which is essential for maintaining normal neuronal function [14]. As neurons age, mitochondrial axonal transport becomes less frequent, which may contribute to the development and progression of neuropathies such as DPN [15]. Mitochondrial axonal transport plays a significant role in ensuring that mitochondria are delivered to distal axons and synapse from the cell body for neuronal function.

ALA, an antioxidant synthesized in mitochondria, serves as a cofactor for mitochondrial oxidative metabolism [16]. It has potent antioxidant effects and is frequently used to prevent and treat DPN. ALA activates AMPK via serine/threonine kinase (LKB1) or calmodulin-dependent protein kinase (CAMKK) [17]. ALA induces mitochondrial dysfunction and cytotoxicity in various tumor cells, where it can activate AMPK, and in some cases, ALA also inhibits invasion through the AMPK–p53 axis [18]. In this study, we conducted both in vivo and in vitro experiments to demonstrate that ALA alleviates the progression of diabetes by regulating mitochondrial axonal transport through the activation of AMPK.

## 2. Materials and methods

### 2.1. Animals

The animal experiments were performed in accordance with the Guiding Principles for the Care and Use of Laboratory Animals published by the National Science and Technology Commission of China. This project was approved by the Ethics Committee of Hebei University of Traditional Chinese Medicine (DWLL202203116). Fifty-five male Sprague‒Dawley rats (7–8 weeks old, 200–240 g) were purchased from Liaoning Changsheng Biotechnology Co., Ltd. (Beijing, China) (SCXK (Liaoning) 2020−0001) and housed in 19 cages in an animal facility under a 12-h/12-h artificial light–dark cycle at a temperature of 22–24°C and a humidity of 40%. The cage bedding was changed daily. At the end of the experiment, the rats were sacrificed using isoflurane overdose. Bilateral sciatic nerves and L4-5 dorsal root ganglia were preserved as samples, with some of them immediately placed in a −80°C freezer for subsequent Western blot analysis, while the remaining tissues were stored in 4% paraformaldehyde for subsequent immunofluorescence staining and other experimental studies.

### 2.2. Induction of diabetes mellitus and drug treatment

Fifteen rats were randomly assigned to the control group and fed a standard diet. The remaining rats were fed a high-carbohydrate/high-fat diet (67% basic feed + 10% lard + 20% sucrose + 2.5% cholesterol + 0.5% sodium cholate; Beijing Science and Australia Cooperative Feed Co., Ltd.) for 6 weeks and then intraperitoneally injected with streptozotocin (STZ) (Sigma‒Aldrich, St. Louis, MO, USA) (30 mg/kg). After 1 week, rats with fasting glucose levels ≥ 16.7 mmol/L were considered diabetic and were further fed a high-carbohydrate/high-fat diet. Diabetic rats were randomly divided into the DPN and ALA groups. After factors such as death and unsuccessful modeling were excluded, 15 animals remained in the DPN group and the ALA group. The rats in the ALA group were administered an ALA (Sigma‒Aldrich, St. Louis, MO, USA) suspension (60 mg/kg/day) by gavage for 12 weeks. The rats in the control group and DPN group were gavaged with an equal volume of distilled water every day for 12 weeks. The motor nerve conduction velocity (MNCV) and paw withdrawal threshold (PWT)of the rats in the ALA group and the DPN group were reduced to varying degrees; therefore, the rats in these groups were considered to have DPN [19].

### 2.3. Analysis of blood glucose levels and weight

After the diabetic rats were identified, fasting glucose levels and body weights were measured and recorded every 4 weeks.

### 2.4. Paw withdrawal threshold (PWT)

PWT was measured by using an electronic von Frey apparatus (type 2390, 90 g probe, 0.8 mm diameter; IITC Life Science, Inc., Woodland Hills, CA, USA) after the 12-week treatment period. The rats were placed on wire mesh, covered with a glass box, and allowed to acclimate to their surroundings for 15 min. A stainless-steel filament was applied vertically to the plantar surface of the hind paw. Rapid paw withdrawal was considered a positive response, and the force that elicited the response was recorded (grams). Paw withdrawal caused by general physical activity was considered a negative response. Measurements were recorded at intervals of 3–5 min. The response of each paw was assessed three times.

### 2.5. Motor nerve conduction velocity (MNCV)

After the 12-week treatment period, the MNCV of the rats were measured. The rats were anesthetized with isoflurane (3% volume) before being fixed in the prone position. The sciatic nerve was exposed through an incision, and the skin between the biceps femoris and semitendinosus muscles was separated from the muscles on the experimental side. Electrodes were implanted at two sites of the sciatic nerve notch approximately 10 mm apart. Platinum wire electrodes were placed directly under the sciatic nerve trunk in the right leg for stimulation, after which the data were recorded. The sciatic nerve was stimulated with a single square-wave pulse (intensity: 1.2 V; width: 1 ms) using an experimental system (BL-420s; Taimeng, Sichuan, China). The MNCV was measured and calculated as follows: MNCV = D/L (m/s).

### 2.6. Histopathological analysis

Five rats were randomly selected from each group, and their sciatic nerves were isolated, fixed in 4% paraformaldehyde for more than 24 h, and then dehydrated using a gradient series of alcohol solutions. After the dehydrated tissue was embedded in paraffin, the tissue was cut into 3 μm thick slices. Afterward, the slices were dewaxed with xylene and stained with hematoxylin–eosin. After dehydration with ethanol, the slices were sealed. Finally, the samples were observed with an optical microscope at a magnification of 400 × . The sciatic nerve axon region was manually selected for each image, and the axon diameter was measured. For each sample, 15 axons were selected to measure the diameter and averaged. Changes in the axon diameter were analyzed using Image-Pro.

### 2.7. Immunofluorescence staining

For immunofluorescence staining, five rats from each group were randomly selected, and sciatic nerves and dorsal root ganglion sections were subjected to routine deparaffinization and rehydration and subsequently subjected to antigen retrieval by incubation with citric acid (0.01 mol/L, pH 6.0) in a microwave oven at approximately 90°C for 20 min. The sections were blocked with nonimmunoreactive serum and incubated with antibodies against KIF5A (Abcam, USA, 1:500), DYNC1I2 (Proteintech, USA, 1:100), p-AMPK (Immunoway, USA, 1:100), and p-CREB (Abcam, USA, 1:100) at 4°C overnight and subsequently incubated with secondary antibodies at 37°C for 1 h. After staining, the sections were incubated with DAPI for nuclear staining and sealed for imaging using an inverted fluorescence microscope (CTS SP8, Leica, Germany). Semiquantitative analysis was performed on the basis of the fluorescence intensity of the cells. The images were analyzed using ImageJ.

### 2.8. Western blot analysis of the expression of related proteins in tissues

Five rats were randomly selected from each group, and their sciatic nerves and dorsal root ganglion tissues were homogenized in RIPA buffer. Protein samples were separated on 10% SDS–PAGE gels and subsequently transferred onto nitrocellulose membranes (Pall Gelman, Ann Arbor, MI, USA). After transfer, the membranes were blocked with 5% skim milk powder. Afterward, the membranes were incubated with antibodies against KIF5A (Abcam, USA; 1:1000), DYNC1I2 (Proteintech, USA; 1:500), AMPK (Immunoway, USA; 1:1000), p-AMPK (Immunoway, USA; 1:1000), CREB (Proteintech, USA; 1:500), and p-CREB (Abcam, USA; 1:500) at 4°C overnight, followed by incubation with a fluorescent dye-conjugated secondary antibody for 1 h at room temperature. A laser infrared scanner (Odyssey, LI-COR, USA) was used to visualize the protein bands.

### 2.9. In vitro cell culture

NSC34 cells were maintained in culture medium supplemented with 10% heat-inactivated fetal bovine serum (FBS) and 1% penicillin and streptomycin at 37°C in an incubator with a humidified atmosphere and 5% CO_2_. NSC34 cells were divided into a control group, a DPN group, and an ALA group. Glucose (50 mmol·L^-1^) [20] and palmitic acid (250 μmol·L^-1^) [21] were added to the DPN group and ALA group. The ALA group was administered 250 μM ALA [22]. The relevant indices were measured 24 h after treatment.

### 2.10. Immunofluorescence staining

Dried and sterilized glass slides were placed in 24-well Petri dishes, the cells were spread evenly on the glass slides in the wells, 4% paraformaldehyde was added, and the plates were incubated for 20 min at room temperature. Afterward, 0.25% Triton X-100 was added and incubated for 15 min for permeabilization, and 10% normal goat serum was added and incubated for 30 min for blocking. The cells were subsequently incubated with primary antibodies against KIF5A (Abcam, USA; 1:300), DYNC1I2 (Proteintech, USA; 1:200), p-AMPK (Immunoway, USA; 1:200), and p-CREB (Abcam, USA; 1:200) overnight at 4°C, followed by the corresponding secondary antibodies at 37°C in the dark for 1 h for fluorescence staining. DAPI was used for nuclear staining. β-Tubulin III staining was used to measure the length of the neurons and axons. After immunofluorescence staining, 15 different neurons were randomly selected from each group of cells, and the axonal length of the neurons was measured using ImageJ.

### 2.11. Western blot analysis of the expression of related proteins in cells

NSC34 cells were homogenized in RIPA buffer, and protein samples were separated on 10% SDS–PAGE gels and transferred onto nitrocellulose membranes (Pall Gelman, Ann Arbor, MI, USA). After transfer, the membranes were blocked with 5% skim milk powder. Afterward, the membranes were incubated with antibodies against KIF5A (Abcam, USA; 1:1000), DYNC1I2 (Proteintech, USA; 1:500), AMPK (Immunoway, USA; 1:1000), p-AMPK (Immunoway, USA; 1:1000), CREB (Proteintech, USA; 1:500), and p-CREB (Abcam, USA; 1:500) at 4°C overnight, followed by incubation with a fluorophore-conjugated secondary antibody for 1 h at room temperature. A laser infrared scanner (Odyssey, LI-COR, USA) was used to visualize the protein bands.

### 2.12. Statistical analysis

SPSS 24.0 (IBM Corp., released 2016, IBM SPSS Statistics for Windows, Version 24.0. Armonk, NY: IBM Corp.) was used to analyze the data. All the data are presented as the means ± standard deviations (M ± SD). Normally distributed data were compared among multiple groups using one-way ANOVA followed by Tukey’s test. Statistical tests were performed using two-sided tests. *P <* 0.05 was considered to indicate statistical significance.

## 3. Results

### 3.1. Rat body weight and fasting blood glucose levels

**Fig 1:**
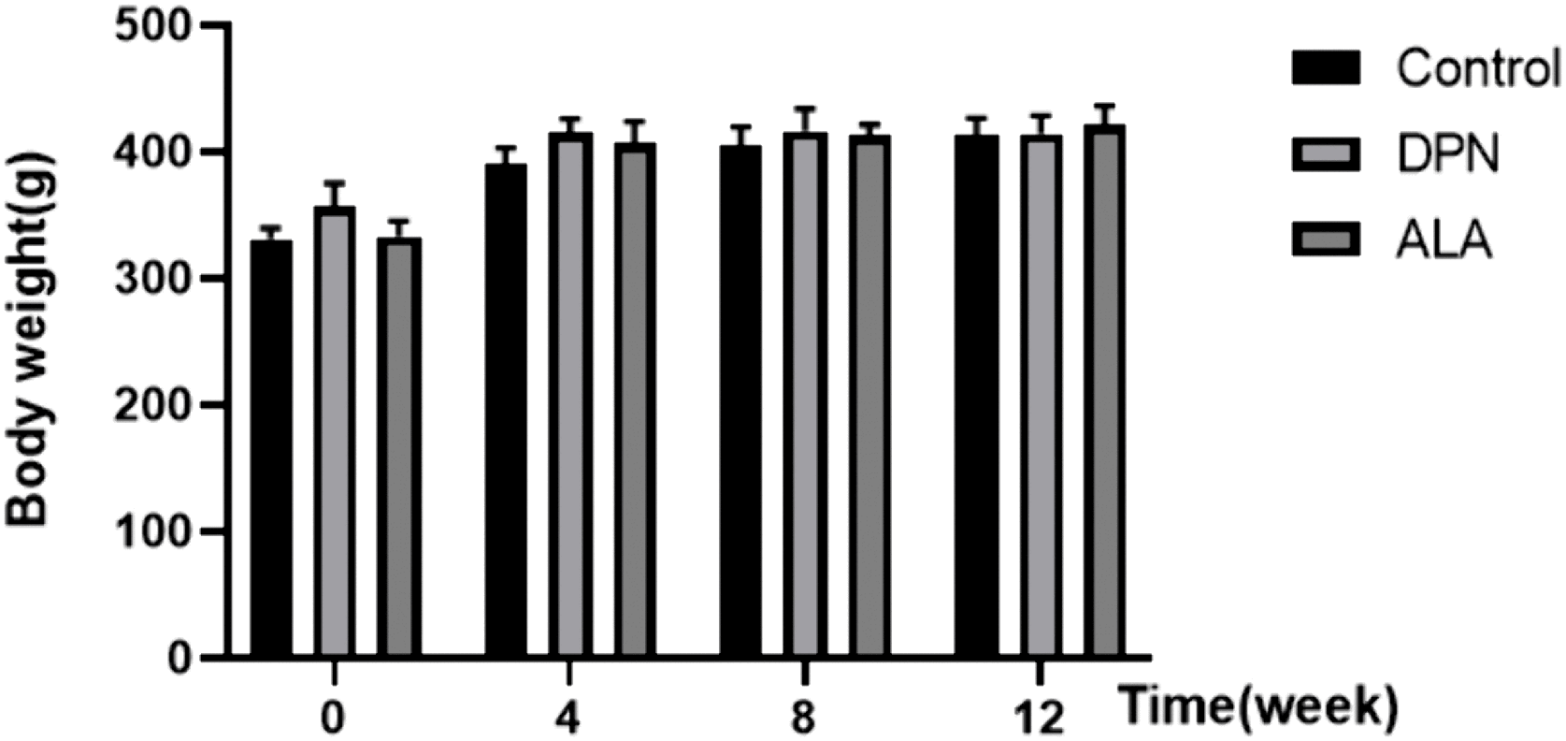
Effects of ALA on the body weight of rats (g). DPN, diabetic peripheral neuropathy; ALA, α-lipoic acid (60 mg/kg/day). The values are presented as the means ± standard deviations; *n =* 15 rats in each group.

**Fig 2:**
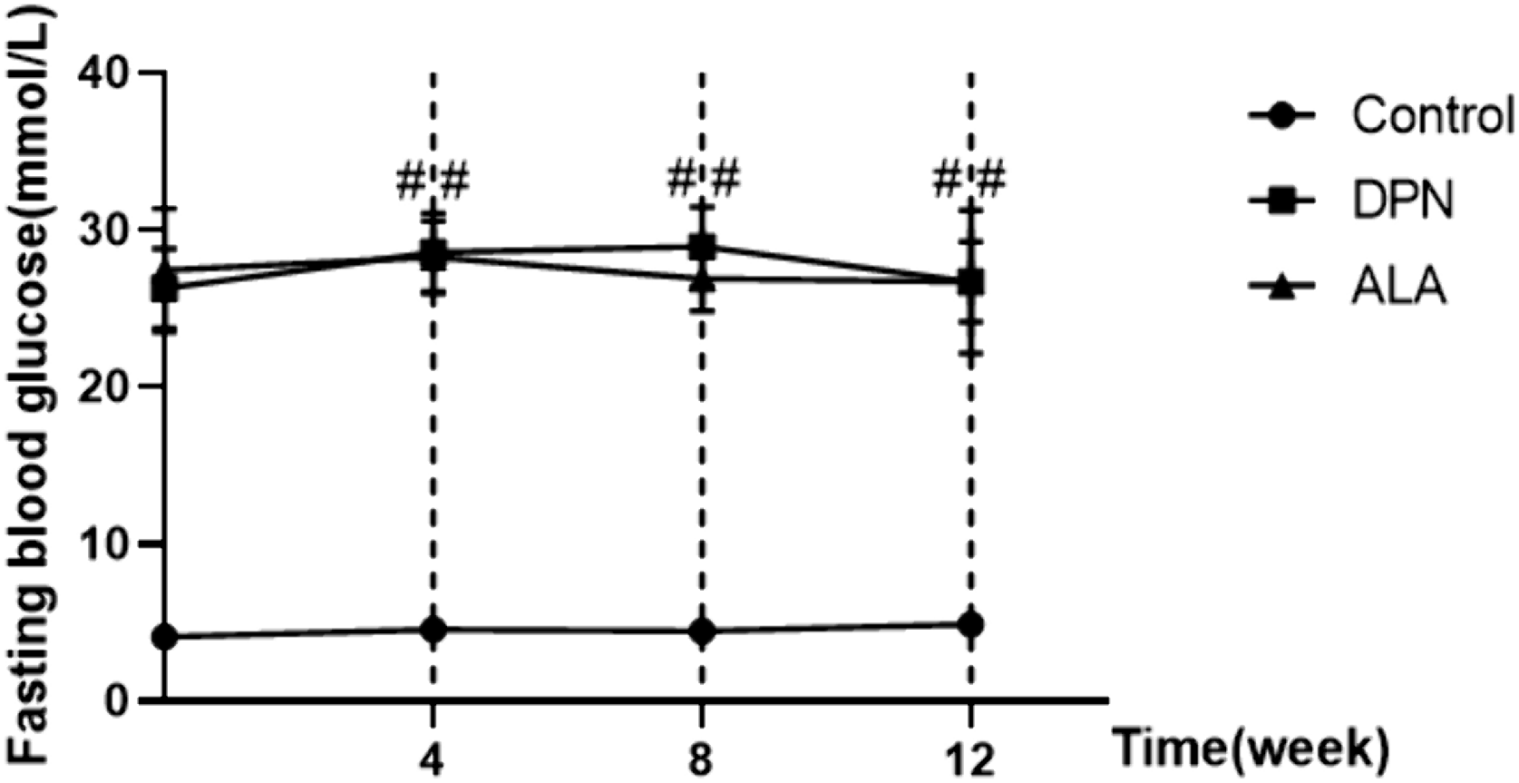
Effects of ALA on the fasting blood glucose levels (mmol/L) of rats. The values are presented as the means ± standard deviations; *n =* 15 rats in each group. ## *P <* 0.01 vs. the control group. One-way ANOVA with Tukey’s multiple comparisons test.

As shown in Fig 1, rats fed a high-carbohydrate/high-fat diet experienced slight weight gain after 4 weeks. However, this difference was not statistically significant. As shown in Fig 2, fasting blood glucose levels were significantly higher in both the DPN and ALA groups than in the control group. No significant difference in fasting blood glucose levels was observed between the DPN group and the ALA group.

### 3.2. Effect of ALA on the paw withdrawal thresholds of rats

**Fig 3:**
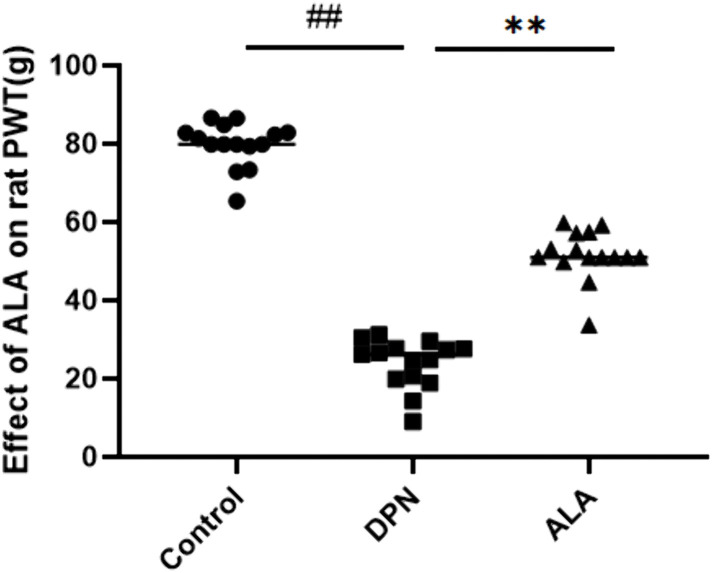
Effect of ALA on the PWT (g) of rats. The values are presented as the means ± standard deviations; *n =* 15 rats in each group. ## *P <* 0.01 vs. the control group; ** *P <* 0.01 vs. the DPN group. One-way ANOVA with Tukey’s multiple comparisons test.

As illustrated in Fig 3, the PWT was significantly lower in the DPN group (*P <* 0.01); however, the PWT was markedly greater in the ALA-treated group than in the DPN group (*P <* 0.01).

### 3.3. Effects of ALA on the motor nerve conduction velocities of rats

**Fig 4:**
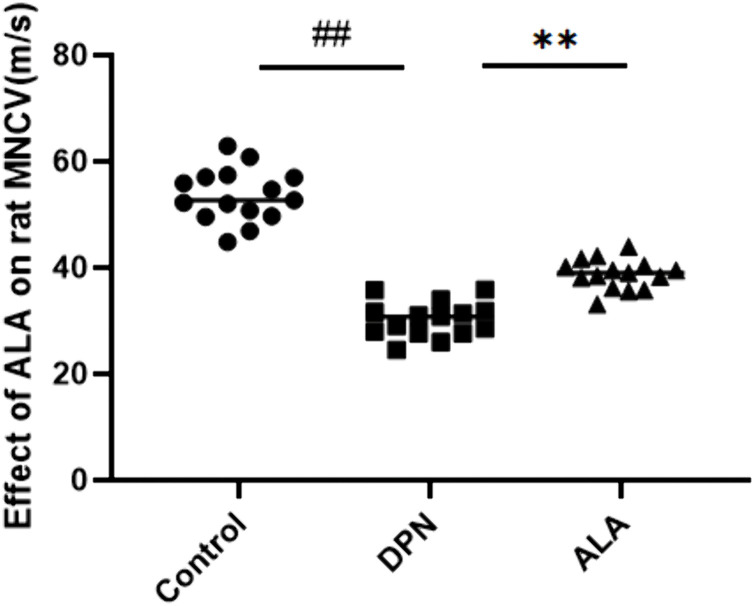
Effect of ALA on the MNCV (m/s) of rats. The values are presented as the means ± standard deviations; *n =* 15 rats in each group. ## *P <* 0.01 vs. the control group; ** *P <* 0.01 vs. the DPN group. One-way ANOVA with Tukey’s multiple comparisons test.

As shown in Fig 4, the MNCV in the DPN group was clearly lower than that in the control group (*P <* 0.01), and the MNCV in the ALA group was significantly greater than that in the DPN group (*P <* 0.01).

### 3.4. Effects of ALA on the morphology of the rat sciatic nerve (H&E staining)

**Fig 5:**
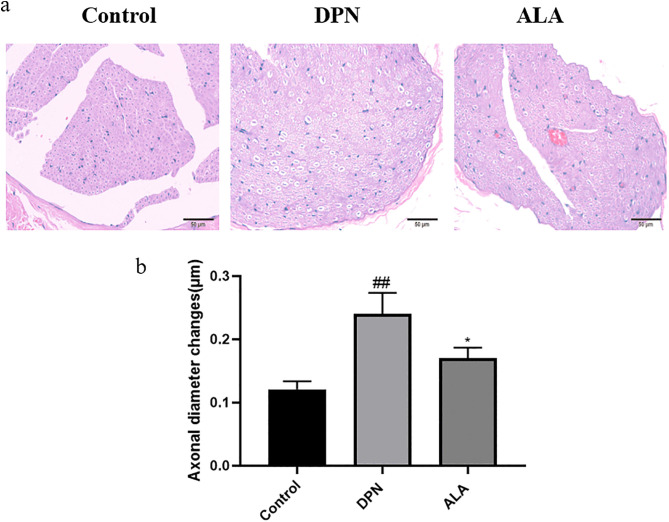
Effect of ALA on the morphology of the rat sciatic nerve (μm) (*n =* 5). a HE staining was performed to observe the morphological changes in the sciatic nerve (scale bar: 50 μm). b Bar plot (means ± SDs) showing changes in the axons of the sciatic nerve. ## *P <* 0.01 vs. the control group; *P < 0.05 vs. the DPN group. One-way ANOVA with Tukey’s multiple comparisons test was used.

As shown in Fig 5a and b, HE staining and microscopy revealed that the structure of the sciatic nerve fibers in the control group was normal, the axons did not exhibit swelling or atrophy, and the structure of the myelin sheath was complete. In the DPN group, a high degree of swelling of nerve axons, partial atrophy, and marked loss of myelin sheaths were observed. Axonal swelling and demyelination were less severe in the ALA group than in the DPN group, and axonal atrophy was not evident.

### 3.5. Effect of ALA on axonal transport in rats

**Fig 6:**
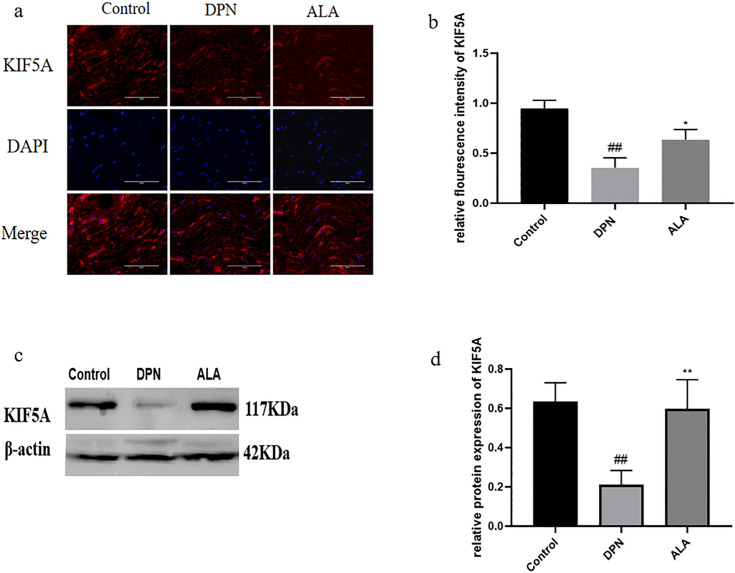
The expression of KIF5A in the sciatic nerve was analyzed by immunofluorescence staining and Western blotting (*n =* 5). a Analysis of KIF5A expression by immunofluorescence staining (scale bar: 50 μm). b Bar plot (means ± SDs) of KIF5A expression detected using immunofluorescence staining. c KIF5A expression was determined by Western blotting. d Bar plot (means ± SDs) of KIF5A expression analyzed using Western blotting. ## *P <* 0.01 vs. the control group; ** *P <* 0.01, *P < 0.05 vs. the DPN group. One-way ANOVA with Tukey’s multiple comparisons test was used.

As shown in Fig 6a, b, c, and d, the expression of KIF5A in the DPN group was lower than that in the control group (*P <* 0.01). Additionally, KIF5A levels were higher in the ALA group than in the DPN group (*P <* 0.05). The trends revealed by Western blotting were consistent with those revealed by immunofluorescence staining.

**Fig 7:**
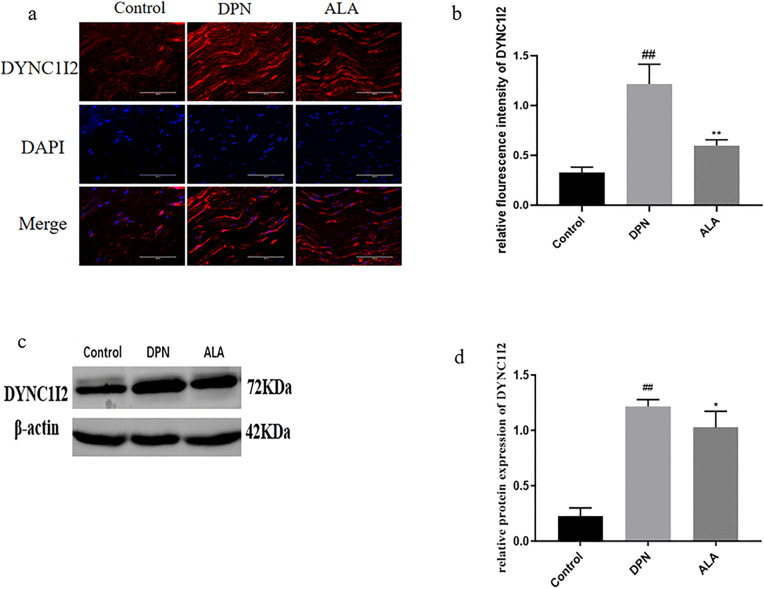
The expression of DYNC1I2 in the sciatic nerve was analyzed by immunofluorescence staining and Western blotting (*n =* 5). a Analysis of DYNC1I2 expression by immunofluorescence staining (scale bar: 50 μm). b Bar plot (means ± SDs) of DYNC1I2 expression detected using immunofluorescence staining. c DYNC1I2 expression determined by Western blotting. d Bar plot (means ± SDs) of DYNC1I2 expression detected by Western blotting. ## *P <* 0.01 vs. the control group; **P < 0.01, * *P <* 0.05 vs. the DPN group. One-way ANOVA with Tukey’s multiple comparisons test was used.

As shown in Fig 7a, b, c, and d, the expression of DYNC1I2 in the DPN group was greater than that in the control group (*P <* 0.01). Additionally, DYNC1I2 (*P <* 0.01) levels were lower in the ALA group than in the DPN group. The trends revealed by Western blotting were consistent with those revealed by immunofluorescence staining.

**Fig 8:**
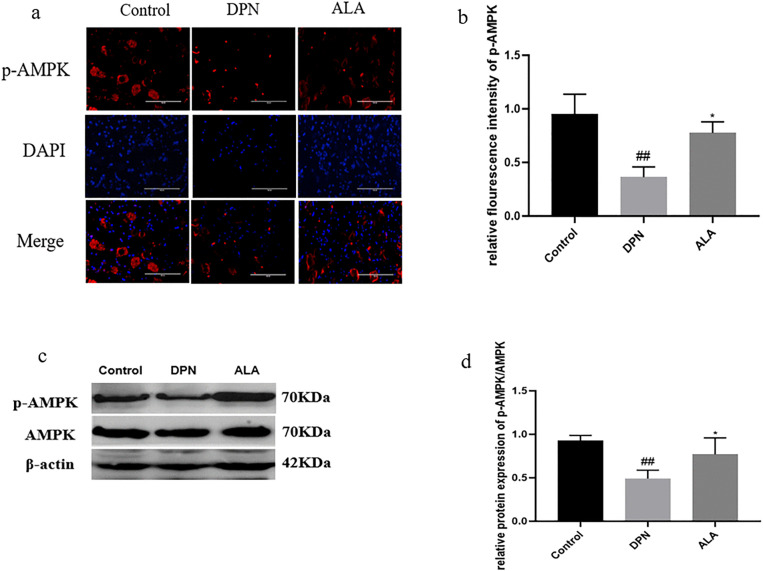
The level of p-AMPK in the dorsal root ganglia was analyzed by immunofluorescence staining and Western blotting (*n =* 5). a Analysis of p-AMPK levels by immunofluorescence staining (scale bar: 50 μm). b Bar plot (means ± SDs) of p-AMPK levels detected using immunofluorescence staining. c p-AMPK and AMPK levels were determined by Western blotting. d Bar plot (means ± SDs) of p-AMPK/AMPK levels detected by Western blotting. ##*P <* 0.01 vs. the control group; *P < 0.05 vs. the DPN group. One-way ANOVA with Tukey’s multiple comparisons test was used.

As shown in Fig 8a, b, c, and d, the level of p-AMPK in the DPN group was lower than that in the control group (*P <* 0.01). Additionally, the p-AMPK level (*P <* 0.05) was higher in the ALA group than in the DPN group. The trends revealed by Western blotting were consistent with those revealed by immunofluorescence staining.

**Fig 9:**
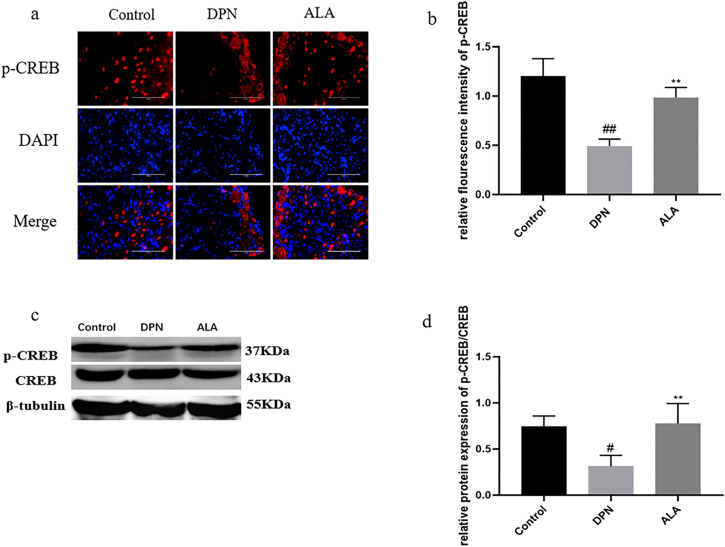
The level of p-CREB in the dorsal root ganglia was analyzed by immunofluorescence staining and Western blotting (*n =* 5). a Analysis of p-CREB levels by immunofluorescence staining (scale bar: 50 μm). b Bar plot (means ± SDs) of p-CREB levels detected using immunofluorescence staining. c Levels of p-CREB and CREB determined by Western blotting. d Bar plot (means ± SDs) of p-CREB/CREB levels detected by Western blotting. ##*P <* 0.01, #*P <* 0.05 vs. the control group; ** *P <* 0.01 vs. the DPN group. One-way ANOVA with Tukey’s multiple comparisons test was used.

As shown in Fig 9a, b, c, and d, the level of p-CREB in the DPN group was lower than that in the control group (*P <* 0.01). Additionally, p-CREB levels (*P <* 0.01) were higher in the ALA group than in the DPN group. The trends revealed by Western blotting were consistent with those revealed by immunofluorescence staining.

### 3.6. Effects of ALA on axonal transport in NSC34 cells

**Fig 10:**
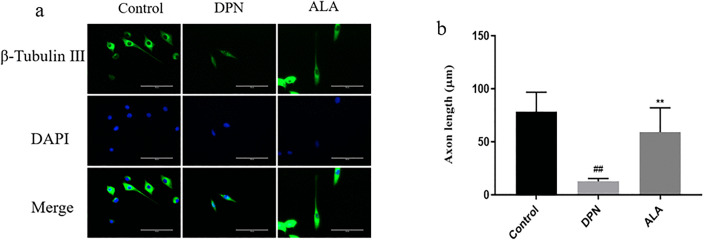
Effect of ALA on changes in neuronal axon length in NSC34 cells. a Analysis of neuronal axon length by immunofluorescence staining (scale bar: 50 μm). b Bar plot (means ± SDs) of the changes in neuronal axon length. ## *P <* 0.01 vs. the control group; ** *P <* 0.01 vs. the DPN group. One-way ANOVA with Tukey’s multiple comparisons test was used.

β-Tubulin III is a neuron-specific tubulin isoform that is almost exclusively expressed in neurons and is involved in neuronal development, axon outgrowth and orientation through the regulation of microtubule dynamics. Therefore, the β-tubulin III antibody can stain the cytoskeleton of neurons, and the axonal length can be measured. As shown in Fig 10a, b, compared with that in the control group, the neuronal axon length in the DPN group was decreased (*P <* 0.01). Additionally, the length of neuronal axons was greater in the ALA group than in the DPN group (*P <* 0.01).

**Fig 11:**
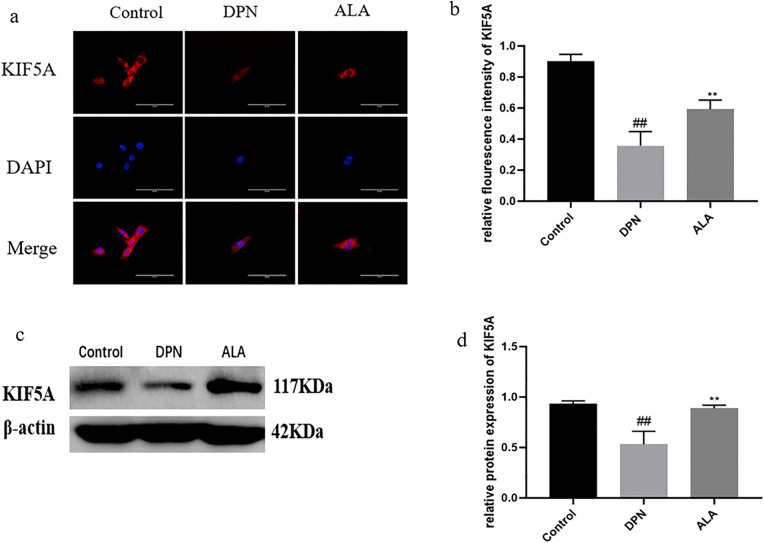
Analysis of KIF5A expression by immunofluorescence staining and Western blotting (*n =* 5). a Analysis of KIF5A expression by immunofluorescence staining (scale bar: 50 μm). b Bar plot (means ± SDs) of KIF5A expression detected using immunofluorescence staining. c KIF5A expression was determined by Western blotting. d Bar plot (means ± SDs) of KIF5A expression detected using Western blotting. ## *P <* 0.01 vs. the control group; **P < 0.01 vs. the DPN group. One-way ANOVA with Tukey’s multiple comparisons test was used.

As shown in Fig 11a, b, c and d, the expression of KIF5A in the DPN group was lower than that in the control group (*P <* 0.01). Additionally, compared with those in the DPN group, KIF5A levels in the ALA group were greater (*P <* 0.01). The trends revealed by Western blotting were consistent with those revealed by immunofluorescence staining.

**Fig 12:**
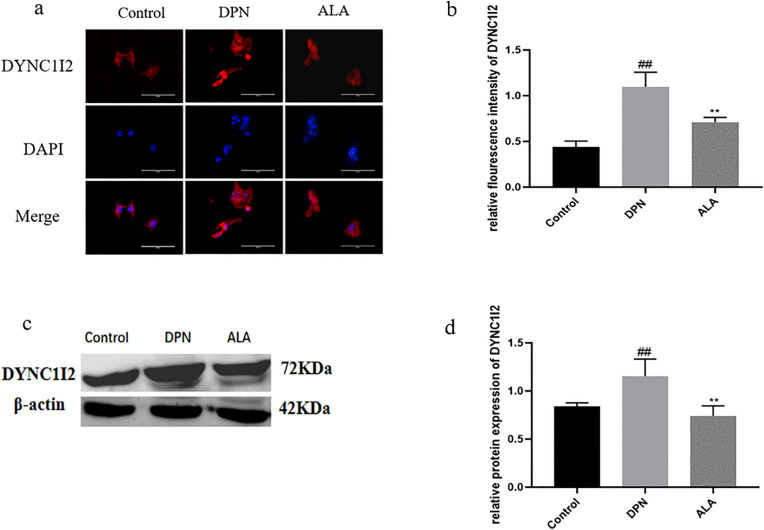
Analysis of DYNC1I2 expression by immunofluorescence staining and Western blotting (*n =* 5). a Analysis of DYNC1I2 expression by immunofluorescence staining (scale bar: 50 μm). b Bar plot (means ± SDs) of DYNC1I2 expression detected using immunofluorescence staining. c The expression of DYNC1I2 determined by Western blotting. d Bar plot (means ± SDs) of DYNC1I2 expression detected using Western blotting. ##*P <* 0.01 vs. the control group; **P < 0.01 vs. the DPN group. One-way ANOVA with Tukey’s multiple comparisons test was used.

As shown in Fig 12a, b, c, and d, the expression of DYNC1I2 in the DPN group was greater than that in the control group (*P <* 0.01). Additionally, DYNC1I2 levels were lower in the ALA group than in the DPN group (*P <* 0.01). The trends revealed by Western blotting were consistent with those revealed by immunofluorescence staining.

**Fig 13:**
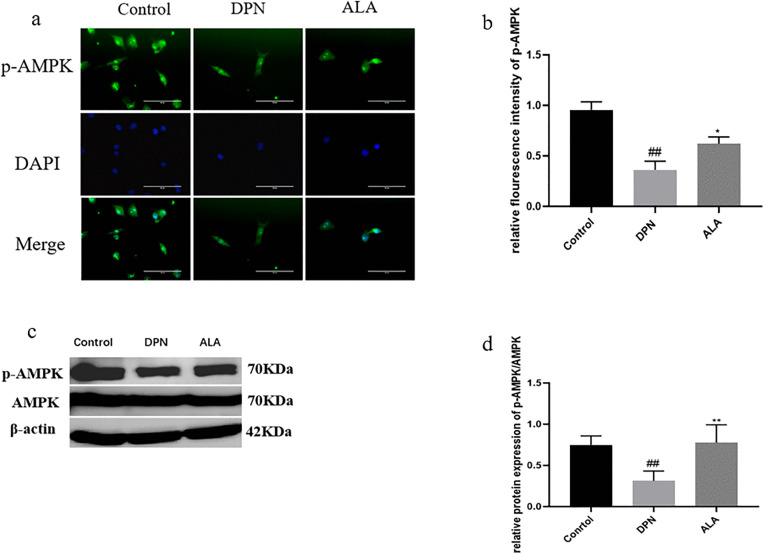
Analysis of p-AMPK levels by immunofluorescence staining and Western blotting (*n =* 5). a Analysis of p-AMPK levels by immunofluorescence staining (scale bar: 50 μm). b Bar plot (means ± SDs) of p-AMPK levels detected using immunofluorescence staining. c p-AMPK and AMPK levels were determined by Western blotting. d Bar plot (means ± SDs) of p-AMPK/AMPK levels detected using Western blotting. ##*P <* 0.01 vs. the control group; **P < 0.01, *P < 0.05 vs. the DPN group. One-way ANOVA with Tukey’s multiple comparisons test was used.

As shown in Fig 13a, b, c, and d, the level of p-AMPK in the DPN group was lower than that in the control group (*P <* 0.01). Additionally, p-AMPK levels were increased in the ALA group compared with those in the DPN group (*P <* 0.05). The trends revealed by Western blotting were consistent with those revealed by immunofluorescence staining.

**Fig 14:**
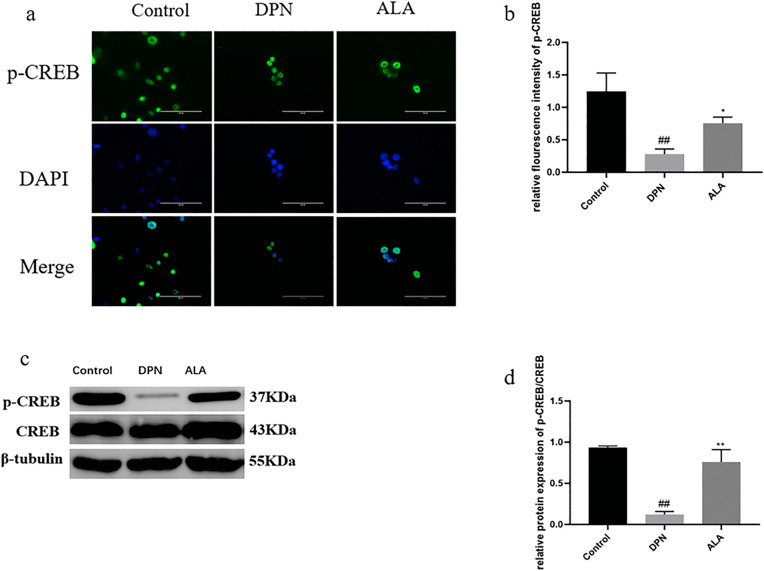
Analysis of p-CREB levels by immunofluorescence staining and Western blotting (*n =* 5). a Analysis of p-CREB levels by immunofluorescence staining (scale bar: 50 μm). b Bar plot (means ± SDs) of p-CREB levels detected using immunofluorescence staining. c Levels of p-CREB and CREB determined by Western blotting. c Bar plot (means ± SDs) of p-CREB/CREB levels detected using Western blotting. ##*P <* 0.01 vs. the control group; **P < 0.01, *P < 0.05 vs. the DPN group. One-way ANOVA with Tukey’s multiple comparisons test was used.

As shown in Fig 14a, b, c, and d, the level of p-CREB in the DPN group was lower than that in the control group (*P <* 0.01). Additionally, p-CREB levels were increased in the ALA group compared with those in the DPN group (*P <* 0.05). The trends revealed by Western blotting were consistent with those revealed by immunofluorescence staining.

## 4. Discussion

Alpha-lipoic acid (ALA) is a naturally occurring compound with multiple biological functions that is widely distributed in animal and plant tissues and has diverse therapeutic potential [23]. ALA, an antioxidant that is soluble in both water and lipids, is known as a ‘universal antioxidant.’ This unique characteristic allows ALA to increase the body’s antioxidant capacity through multiple mechanisms, including directly scavenging free radicals, regenerating endogenous antioxidants, chelating metal ions, and regulating inflammatory responses [24]. Diabetic peripheral neuropathy (DPN) is becoming increasingly common, causing significant suffering for patients. However, there is currently no effective cure. Current guidelines recommend treatments for DPN mainly to relieve symptoms rather than to alter the disease itself. Among mechanism-based therapies, ALA holds a unique position as a universal antioxidant and is essential for every cell in the body [25].

MNCV is an important electrophysiological index for the assessment of peripheral nerve function and is closely related to DPN [26]. In DPN, oxidative stress destroys the lipid structure of the myelin sheath and allows infiltration by the inflammatory cytokines tumor necrosis factor-α (TNF-α) and interleukin-6 (IL-6), resulting in demyelination and nerve blockage [27]. Patients with DPN often present with analgesia, including hyperalgesia or hypoalgesia, and the PWT can reflect these changes by enabling the quantification of mechanical pain thresholds [28]. Abnormal pain perception in individuals with DPN is closely related to ion channel remodeling and imbalanced nerve growth factor signaling after nerve injury, impaired survival and differentiation of sensory neurons, aggravated pain sensitization, and decreased pain thresholds in rats [29]. Compared with the control group, the DPN group had significantly lower MNCV and PWT. MNCV and PWT were significantly greater in the ALA group than in the DPN group. These findings suggest that ALA can repair sensory nerve damage and reestablish pain regulatory mechanisms to alleviate hyperalgesia symptoms in DPN rats.

Pathological changes in the sciatic nerve are key indicators for evaluating DPN injury and intervention effects [30]. HE staining revealed that the sciatic nerve fiber structure in the control group was normal, the axons were not swollen or atrophied, and the myelin sheath was intact. These findings reflect the morphological characteristics of normal nerve fibers: axons maintain conduction function, and myelin sheaths ensure efficient signal transmission. The axons were highly swollen and partially atrophied in the DPN group, with a significant loss of myelin. These alterations are directly associated with nerve conduction disorders, such as paresthesias and decreased motor function, in DPN patients. The pathological changes in the ALA group were distinct from those in the DPN group: the degree of axonal swelling and demyelination was significantly reduced, and axonal atrophy was not obvious. These results indicated that ALA protected axons and alleviated sciatic nerve injury in rats with DPN. As a powerful antioxidant, ALA has received widespread attention because of its role in metabolic syndrome, diabetes, and neurodegenerative diseases [31–34]. ALA and its reduced form dihydrolipoic acid can quench superoxide anions, hydroxyl radicals, etc., and reduce mitochondrial oxidative stress and damage [24]. In this study, ALA also exerted a significant neuroprotective effect.

KIF5A, a protein involved in anterograde mitochondrial transport in neurons, plays a role in DPN [35]. Studies have shown that mice in which KIF5A is knocked out die shortly after birth and have severe axonal damage [36]. In the pathological environment, the number of axonal terminals of aging or damaged mitochondria is increased, mitochondria need to be transported back to neurons through axons, and DYNC1I2 interacts with microtubules to ensure the ability of damaged mitochondria to be transported in the reverse direction within neurons and maintain the direction of transport [37]. Our results revealed that KIF5A expression was significantly lower in the DPN group than in the control group and was increased after ALA administration. ALA may exert neuroprotective effects by promoting anterograde mitochondrial transport. In contrast, DYNC1I2 expression tended to decrease, with DYNC1I2 expression increasing in the DPN group but decreasing in the ALA group. These results indicated that ALA administration could reduce mitochondrial damage at nerve terminals and weaken retrograde transport. This process may constitute an additional pathway through which ALA achieves neuroprotective effects in addition to its antioxidant properties.

AMPK is a cellular energy sensor expressed by a variety of organs and is closely related to metabolic diseases such as cancer, obesity and diabetes [38]. The role of the AMPK pathway in the prevention and treatment of diabetic complications has received increasing attention in recent years. Studies have shown that AMPK expression is decreased under DPN conditions [39]. Blood glucose levels are closely causally related to DPN, and chronic hyperglycemia is the main cause of DPN [40]. Research has shown that ALA can increase insulin sensitivity by promoting the phosphorylation and subsequent activation of AMPK, which contributes to lowering blood glucose levels [41]. However, our results revealed that blood glucose levels were significantly higher and remained higher in the DPN and ALA groups than in the control group, and a slight but not statistically significant decrease in blood glucose levels was observed in the ALA group compared with the DPN group, suggesting that ALA may not exert a neuroprotective effect through glucose-lowering effects. CREB is the downstream target of AMPK, and as a crucial nuclear transcription factor, it can increase the level of kinase protein subunits when activated [42]. Numerous studies have described a synergistic relationship between AMPK and CREB and their involvement in adaptive responses to cellular energy stress. In the AMPK/CREB metabolic control network, p-AMPK can activate CREB, thereby regulating the expression of the anterograde axon mitochondrial transport protein KIF5A [5,11]. Our results revealed that p-AMPK and p-CREB levels were significantly lower in the DPN group than in the control group, suggesting that p-AMPK activation may be disrupted in the context of DPN, resulting in an impaired mitochondrial energy supply. The levels of p-AMPK and p-CREB were increased after ALA administration in rats, suggesting that the promotion of mitochondrial trafficking by ALA may be related to AMPK/CREB signaling, thereby alleviating peripheral nerve damage in rats with DPN. However, to date, the relationship between AMPK activation and DYNC1I2 expression has not been reported, and this relationship should be further explored in future studies. Research has also indicated that ALA can inhibit autophagy in vascular smooth muscle cells in diabetic patients through the AMPK/mTOR pathway [43]. The neuroprotective effect of ALA and its correlation require further verification.

In in vitro experiments, NSC34 cells were cultured in a high-glucose and high-fat environment. Compared with other cell types, the NSC34 cell line is characterized by long axons and can respond more strongly to axon-related damage and changes in mitochondrial transport. The results of our in vitro experiments are consistent with those of the animal experiments, suggesting that ALA regulates the anterograde transport of axonal mitochondria, which may be related to AMPK/CREB signaling, increasing the energy supply to the end of axons and ultimately playing a neuroprotective role.

However, some limitations remain in these experiments, such as the use of a single ALA dose, the lack of a positive control, and the lack of direct inhibition experiments to test the causal effects of this pathway, which will be gradually remedied in future experiments. In addition, these experimental results should be interpreted with caution when they are generalized to human patients with diabetic peripheral neuropathy because of the inevitable physiological and anatomical differences between rats and humans.

In summary, the results of this study suggest that ALA may regulate mitochondrial transport by activating AMPK, effectively alleviating DPN-related symptoms. ALA has been used to treat DPN and has achieved good results in clinical practice [44,45]. The results of these studies provide more adequate evidence for the pharmacological mechanism of ALA in DPN. However, ALA is mostly used for the short-term treatment of DPN, and future studies on the chronic efficacy and safety of α-lipoic acid are needed. In conclusion, the identified negative effects of DPN highlight the need for targeted interventions, and in the future, we will pay more attention to the specific mechanisms of action, such as mitochondrial dysfunction, oxidative stress, the inflammatory response, and neurotrophic vascular injury, involved in the occurrence and development of DPN and their interconnections and develop new interventional drugs, such as modulators of specific signaling pathways, or treatment technologies with potential application value, such as stem cell therapy and gene therapy. We will validate the effectiveness and safety of new interventions, optimize treatment options, and provide more personalized and quality treatment options for patients with DPN, with the goal of minimizing the negative effects of DPN and improving patient prognosis and quality of life.

## 5. Conclusion

The results of this study suggest that ALA alleviates peripheral nerve injury in diabetic rats by promoting the anterograde axonal transport of mitochondria, which may be related to AMPK/CREB signaling.

## Supporting information

S1 File S1_raw_images. (PDF)
