# Supplementary material for: Effect of ALA on preventing diabetic peripheral neuropathy in rats through mitochondrial axonal transport
Source: PLoS One. 2026 Apr 1;21(4):e0346297. doi: 10.1371/journal.pone.0346297 (PMC13042701; doi:10.1371/journal.pone.0346297)

Dear Editor,

Thank you for your careful review of our manuscript and your valuable suggestions. In this experiment, the original western blot images we provided contained 4 or 5 lanes, while only 3 lanes were shown in the article. We would like to provide a special clarification: The 3 lanes presented in the article are all core experimental group samples from the formal study. The fourth or fifth lane in the original gel image corresponds to exploratory lanes from preliminary experiments. These samples do not belong to the formal experimental groups of this study, and the related data were not included in the results analysis or statistics of this study. Therefore, they were not shown in the article. The purpose of this exclusion is to avoid irrelevant information from the preliminary experiments interfering with the presentation of the core experimental results, and to ensure that the figures are concise and targeted.

All western blot bands shown in this study were reasonably cropped only to remove unrelated blank areas; the quantification of the bands (using ImageJ software) was based solely on the first three valid lanes of the formal experiment. Moreover, the related experiments were repeated multiple times independently, yielding stable and reliable data. To ensure transparency of the research and traceability of the experimental results, we have provided the complete original western blot images containing all 4 or 5 lanes for review.

We ensure that all experimental data in this study are authentic and complete, with no inappropriate selection, and the related materials fully support the above explanation.

The data has been uploaded to Mendeley Data, and the DOI is 10.17632/pxtnf9c92f.1

Marker：Thermo Scientific,USA,26616

Fig. 6
KIF5A

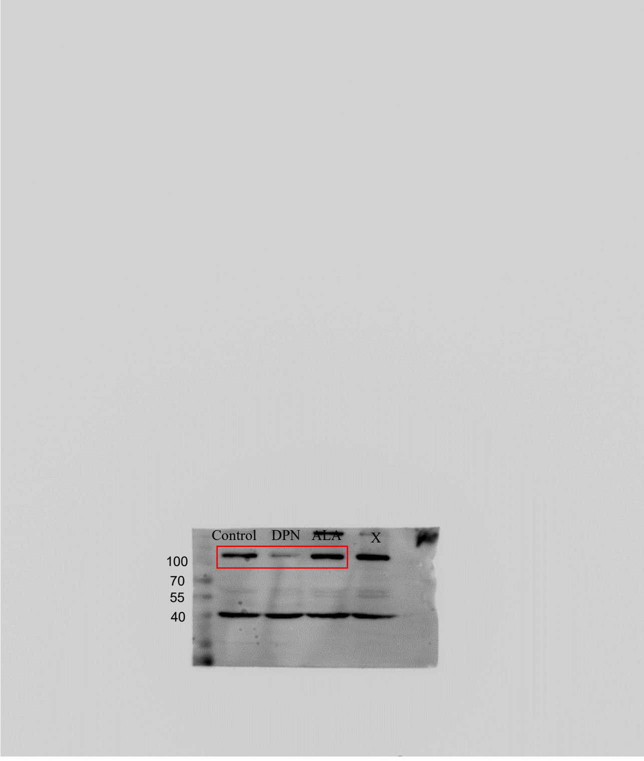

β-actin

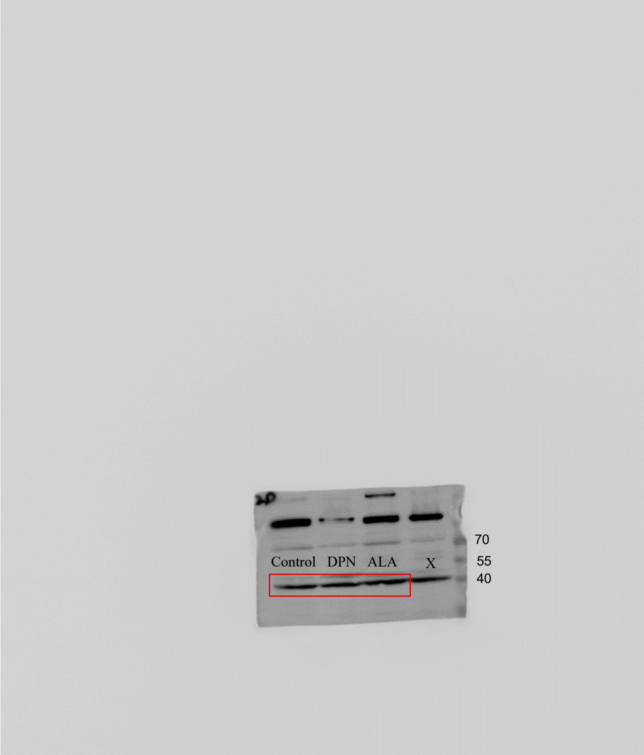

Fig. 7
DYNC1I2

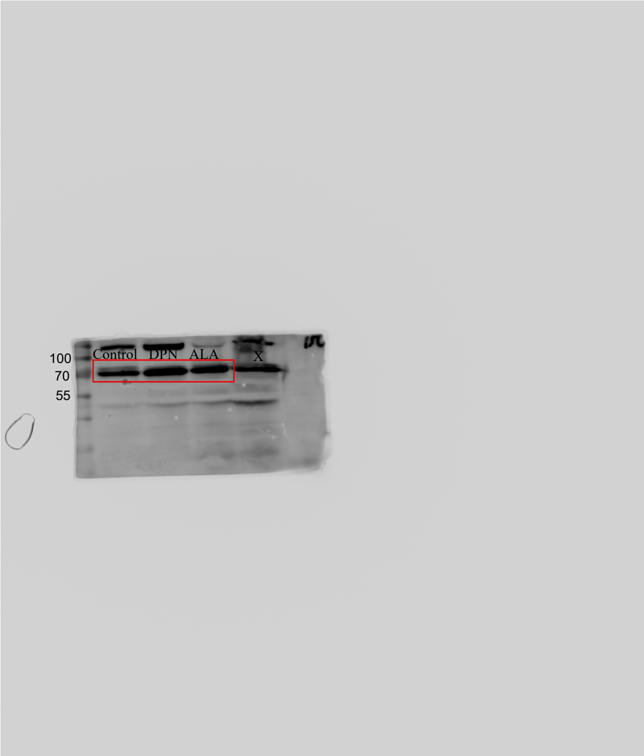

β-actin

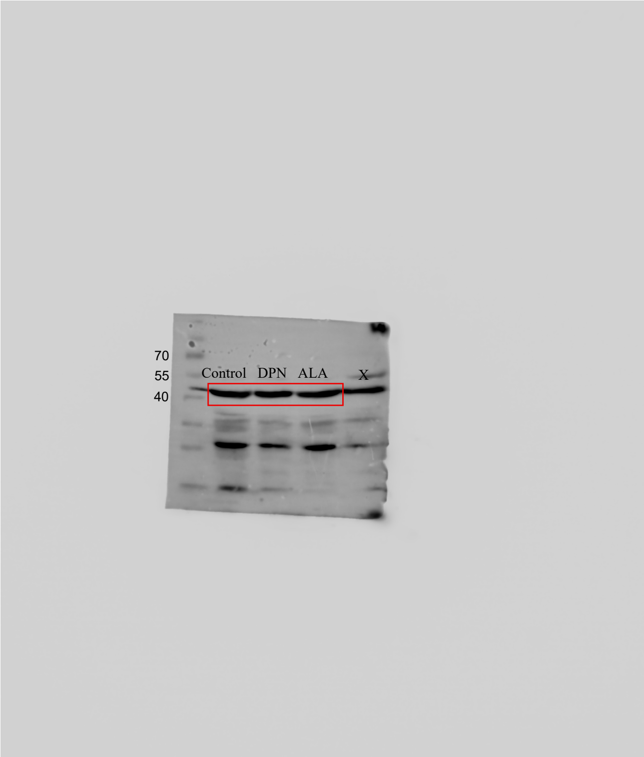

Fig. 8
p-AMPK

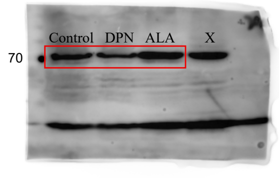

AMPK

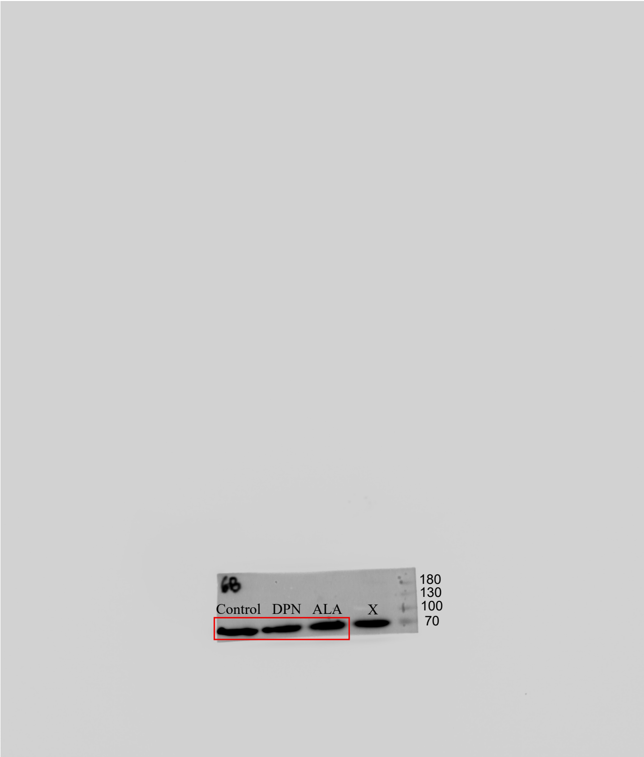

β-actin

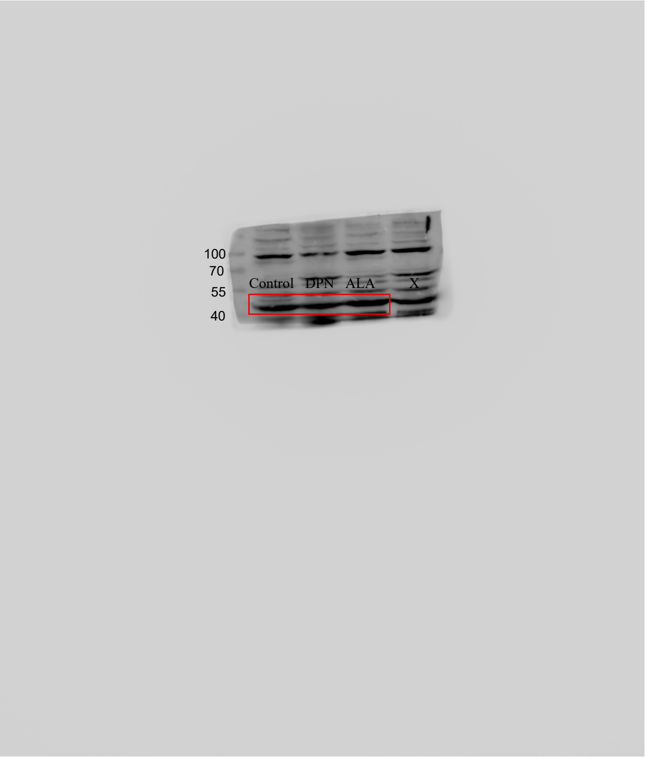

Fig. 9

p-CREB

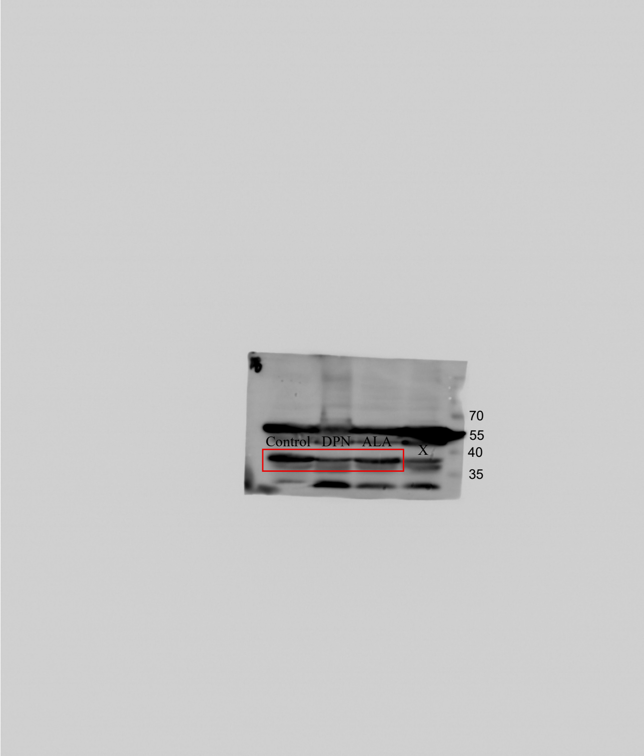

CREB

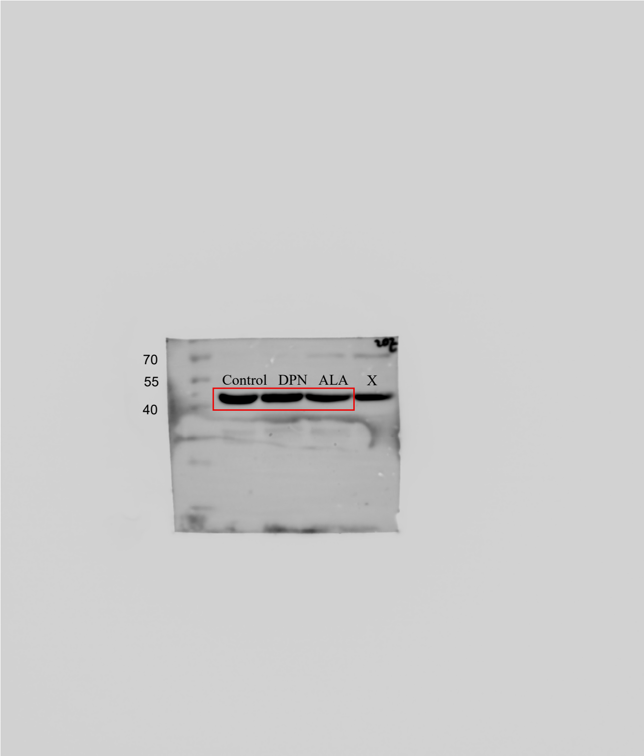

β-tubulin

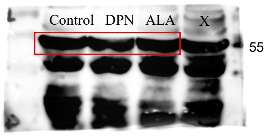

Fig. 11
KIF5A

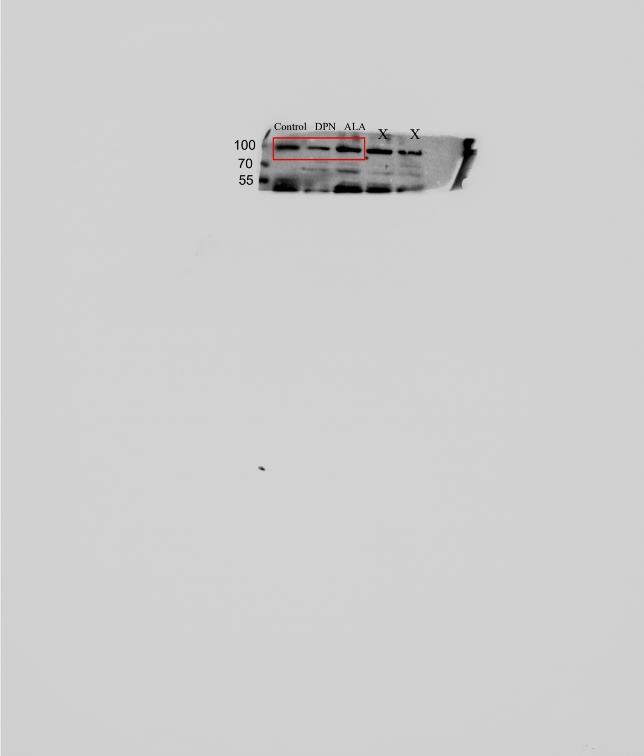

β-actin

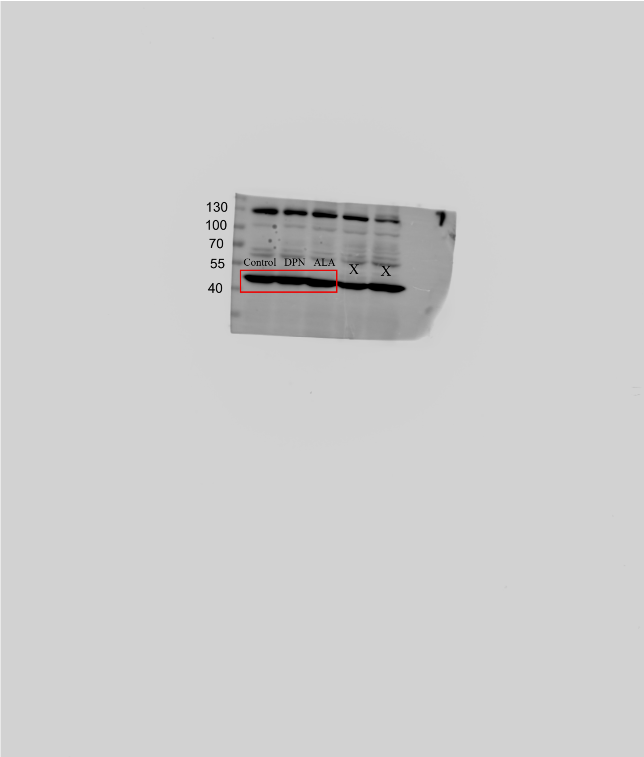

Fig. 12

DYNC1I2

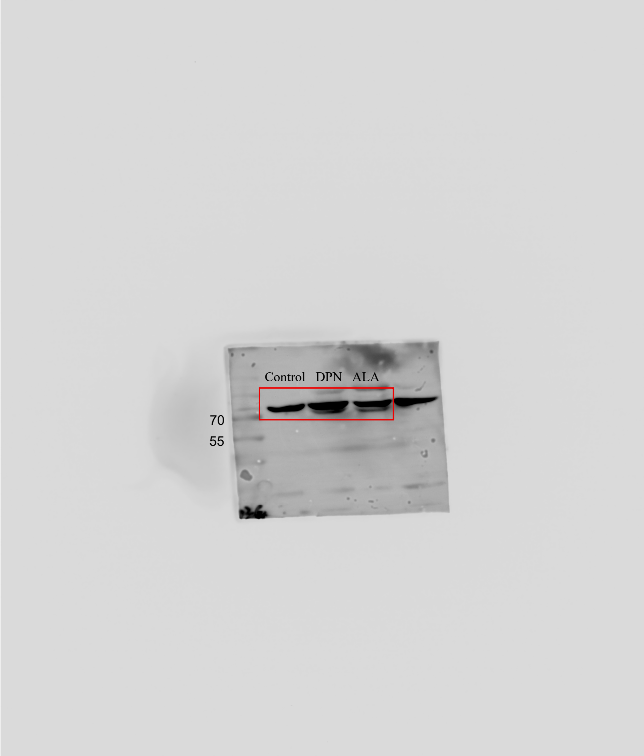

β-actin

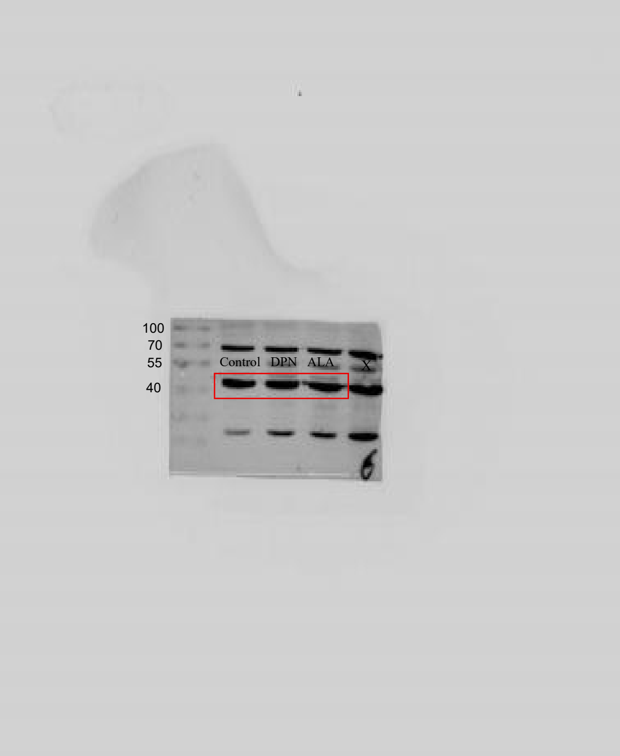

Fig. 13

p-AMPK

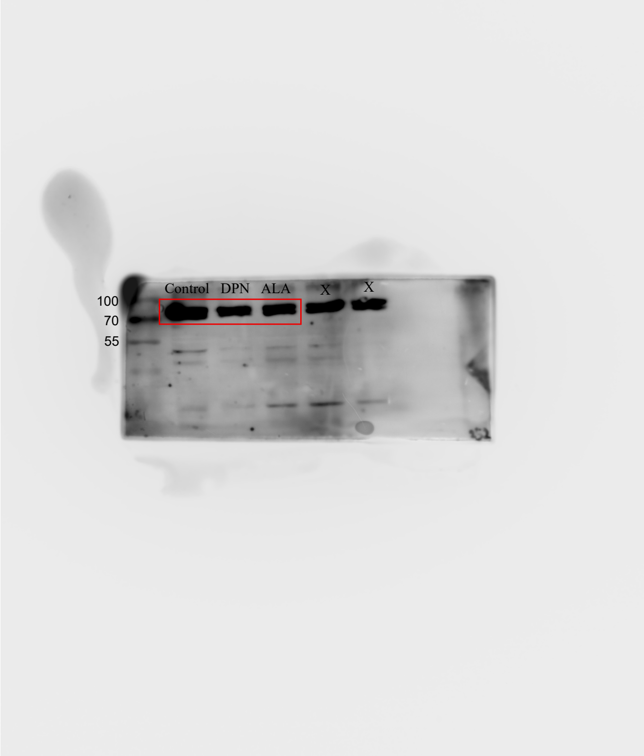

AMPK

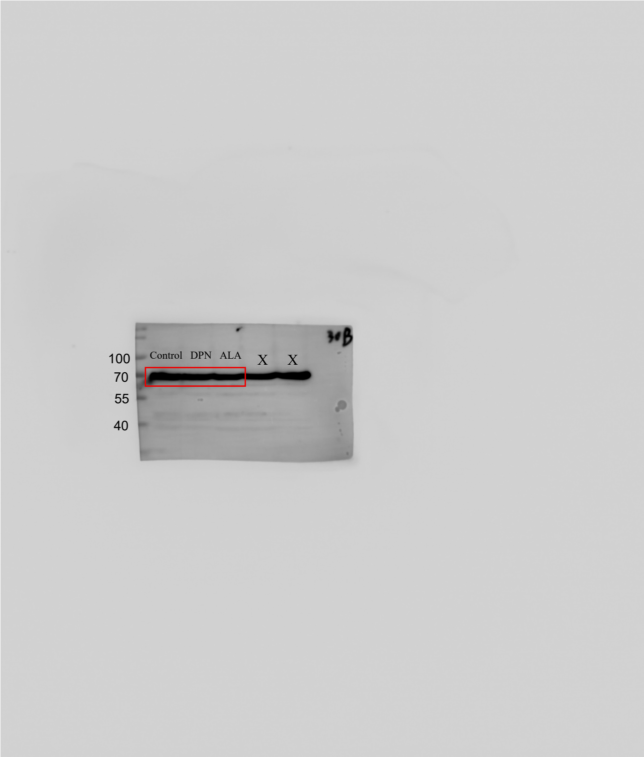

β-actin

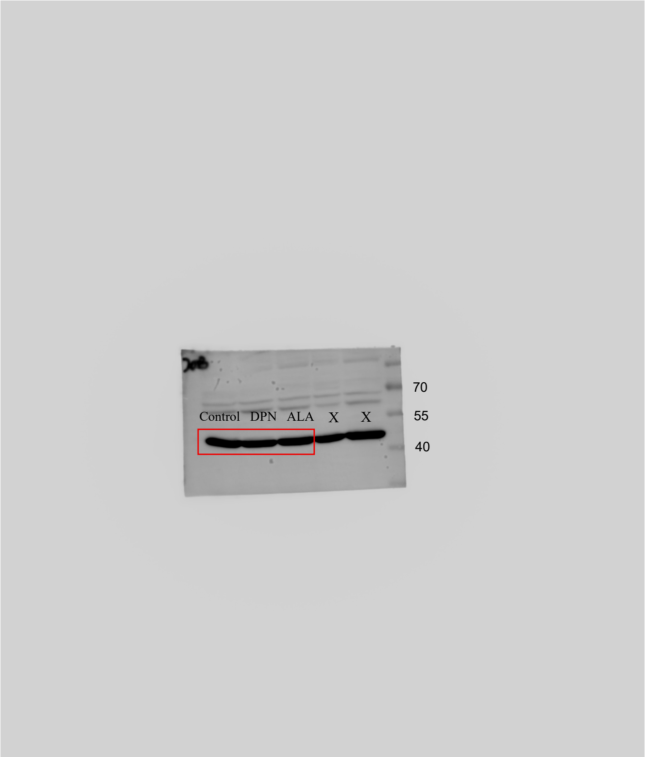

Fig. 14
p-CREB

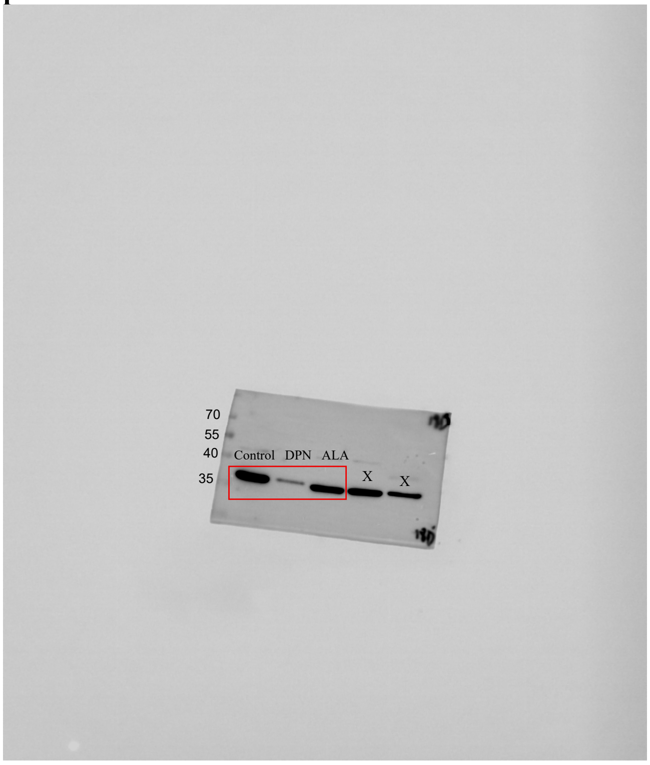

CREB

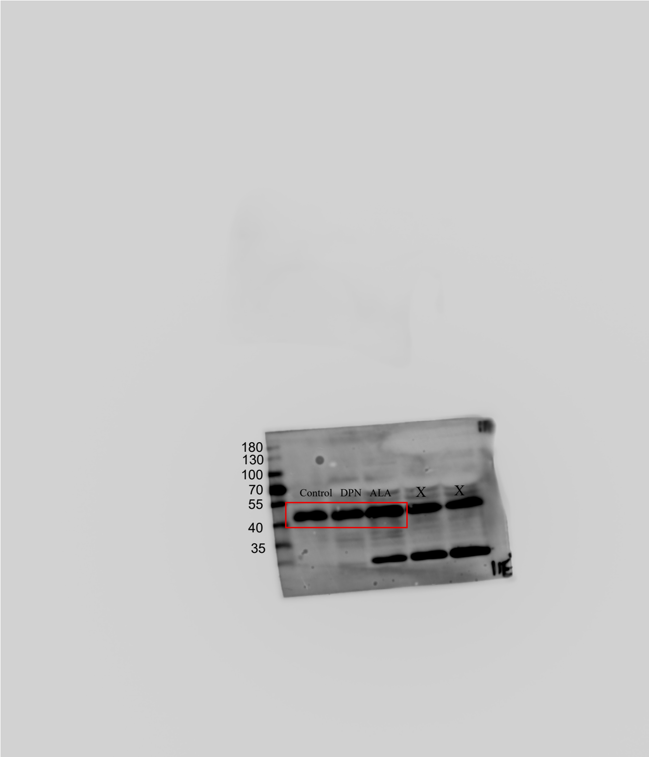

β-tubulin

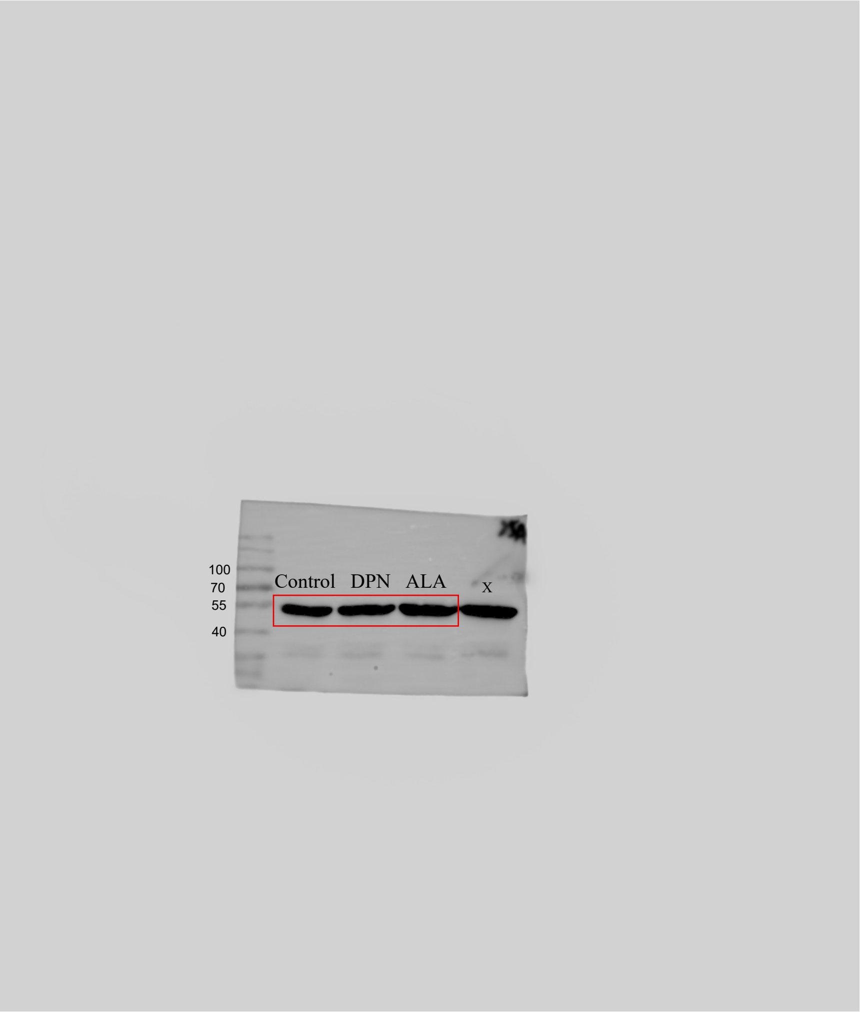

Supplement: S1 File — (PDF) [file pone.0346297.s001.pdf]
